# The dominance of the private sector in the provision of emergency obstetric care: studies from Gujarat, India

**DOI:** 10.1186/s12913-016-1473-8

**Published:** 2016-07-07

**Authors:** Mariano Salazar, Kranti Vora, Ayesha De Costa

**Affiliations:** Department of Public Health Sciences, Karolinska Institutet, Tomtebodavägen 18a, Widerströmska Huset, 171 77 Stockholm, Sweden; Department of Reproductive and Child Health, Indian Institute of Public Health, Gandhinagar, Ahmedabad, Gujarat India

**Keywords:** EmOC, India, Gujarat, Facility-based

## Abstract

**Background:**

India has experienced a steep rise in institutional childbirth. The relative contributions of public and private sector facilities to emergency obstetric care (EmOC) has not been studied in this setting. This paper aims to study in three districts of Gujarat state, India:(a) the availability of EmOC facilities in the public and private sectors; (b) the availability and distribution of human resources for birth attendance in the two sectors; and (c) to benchmark the above against 2005 World Health Report benchmarks (WHR2005).

**Methods:**

A cross-sectional survey of obstetric care facilities reporting 30 or more births in the last three months was conducted (*n* = 159). Performance of EmOC signal functions and availability of human resources were assessed.

**Results:**

EmOC provision was dominated by private facilities (112/159) which were located mainly in district headquarters or small urban towns. The number of basic and comprehensive EmOC facilities was below WHR2005 benchmarks. A high number of private facilities performed C-sections but not all basic signal functions (72/159). Public facilities were the main EmOC providers in rural areas and 40/47 functioned at less than basic EmOC level. The rate of obstetricians per 1000 births was higher in the private sector.

**Conclusions:**

The private sector is the dominant EmOC provider in the state. Given the highly skewed distribution of facilities and resources in the private sector, state led partnerships with the private sector so that all women in the state receive care is important alongside strengthening the public sector.

## Background

Emergency obstetric care (EmOC) is defined as the services “*necessary for the treatment of complications that arise during pregnancy and childbirth*” [1]. EmOC provision is key to reducing maternal mortality [1] as mothers are at most risk of death from serious complications that arise unpredictably during childbirth [2].

Evidence has shown that skilled birth attendants in supportive environments can save lives by detecting and treating obstetric complications [3]. Therefore, a facility-based childbirth strategy, i.e. one in which women are encouraged to give birth in a facility, has been recommended and adopted by many low-and middle-income countries to provide women access to EmOC in an enabling environment [4].

Some countries in South Asia that have experienced high maternal mortality, have over the last decade introduced large scale programs to promote in-facility childbirth [5]. India introduced similar programs in 2005 which resulted in a steep rise in institutional delivery proportions between 2005 and 2012 from 39 to 74 % [6]. In line with national trends, institutional birth proportions in Gujarat, a large state in the west of India, also rose from 40.7 % in 2001 to 89 % in 2010. By 2010, a significant proportion (>60 %) of in-facility births occurred in for-profit private sector facilities [7]. Despite the sharp rise in facility births, and the contribution of the private sector to this, there are no reports studying the availability of EmOC in the state and the relative contributions of public and private sector facilities to this care. This study reports on the above in three districts of Gujarat state. Further, we also look at the availability and distribution of human resources for birth attendance in the public and private sectors. We assess the availability of EmOC and human resources for its provision against 2005 World Health Report [8] benchmarks.

## Methods

### Setting

The study was conducted in Gujarat state, India. Located in the northwest and divided in 26 districts, it has a population of 60.4 million, 57 % rural of which is rural and 33 % live below the poverty line [9]. The maternal mortality ratio (MMR) in 2012 was 122 per 100000 livebirths, which was lower than India’s average for the same year (178 per 100000 livebirths) [10]. The three districts included in this study were purposively selected to represented different areas of the state. They differ on key socio-demographic indicators. They also represent the differences in socioeconomic level seen across the state. Characteristics of the districts are shown in Table 1.

**Table 1:** Characteristics of the study districts

Characteristics	Dahod	Sabarkantha	Surendranagar	Gujarat
Population (2011)^a^	2,127,086	2,428,589	1,756,268	60,383,628
Crude birth rate x 1000 population (2011)^c^	30.2	28	23	22.7
Proportion rural population (2011)^a^	91 %	85 %	72 %	57.4 %
Proportion literate population^a^	58.8 %	75.8 %	72.1 %	79.3 %
Proportion BPL population^b^	71.6 %	32.9 %	46.5 %	40.3 %

^a^Sample Registrar of India (2011). Districts of Gujarat. from http://www.census2011.co.in/census/state/districtlist/gujarat.html. Socio Economic Survey 2002–03. Add-on lists 2008–09 [database available online]. ^b^Commissionerate of Rural Development, Gujarat. http://ses2002.guj.nic.in/. Accessed 15 January 2015. ^c^Vital statistics Division Government of Gujarta. Civil Registration System in Gujarat, Annual Statistical Report 2010. Gandhinagar, 2011

Obstetric care in the state is provided by both public and private health facilities. While public facilities provide services that are formally free of charge, the private sector operates mainly on the basis of out-of-pocket payments [11]. The public sector consists of facilities organized at three levels of care: 1.a single district hospital providing specialist care located at the district headquarter city, 2. sub-district hospitals and community health centers located in smaller towns (some have few beds for admission) 3. primary health care centers and sub-centers further into the rural areas. Childbirth in public facilities is formally free of charge to the user.

The private health sector providing in-patient childbirth care comprises a number of independent facilities, of varying sizes, often owned and run by qualified obstetrician-gynecologists. While these are not formally organized into levels of care, they could be considered to provide secondary level obstetric care, i.e. have fully functional labor rooms and operating rooms for cesarean sections and other procedures. These private facilities operate on the basis of out-of-pocket payments made at the point of care.

#### Design

A cross-sectional facility-based study was conducted (June 2012 - April 2013). A list of all public/private obstetric care facilities (*n* = 1292) was obtained from Gujarat State Health Department and from Gujarat chapter of the Federation of Obstetric and Gynecological Society of India (professional body of obstetrician-gynecologists). The list obtained was verified at the district level.

#### Data collection

An initial screening recording the number of births conducted in all facilities (*n* = 1292) was performed. The initial screening showed that 77.4 % (*n* = 1000) of all facilities reported no childbirths in the last three months, 10.3 % (*n* = 133) had between one and 29 births and 12.3 % (*n* = 159) had 30 or more births. The total number of births was 29597. Further analysis showed that mean number of births for the last two categories were 9 (SD 7.3) and 178 (SD 191) respectively. In this paper, we chose to include only facilities with 30 or more births in the last three months, as 95.6 % of all births occurred in these facilities (28303/29597).

Facilities meeting the criterion for inclusion (*n* = 159) were further assessed using a modified version of the Averting Maternal Death and Disability questionnaire [12], which elicited information on the performance of EmOC signal functions in the last three months. These included seven basic EmOC (BEmOC) signal functions (administration of parenteral antibiotics, uterotonic drugs, parenteral anticonvulsants, manual removal of the placenta, removal of retained products of conception, assisted vaginal delivery, and neonatal resuscitation), and two comprehensive EmOC (CEmOC) functions (caesarean sections [CS] and blood transfusions) [1]. Facilities were classified into four groups based on the reported performance of signal functions: a. BEmOC (those providing all seven basic functions), b. Less-than-BEmOC (facilities not performing all the seven basic functions), c. CEmOC (those performing all nine functions). In addition to these three well established categories, we created an additional category, given the large number of facilities with this particular permutation of EmOC functions in our setting. This fourth category comprised facilities which regularly performed CS but did not perform all the seven BEmOC functions. This fourth category has been labeled d. Less-than-BEmOC + CS. Reasons for non-performance the signal functions in the last 3 months were also recorded and categorized: training, supplies/drug, management, and policy issues [1]. The number of births (vaginal and abdominal) conducted in the previous three months were also recorded from facility registers.

In addition, trained research assistants interviewed the main physician/ administrator in the facility to collect information on facility ownership (public and private), total bed strength, and human resources (obstetricians, anesthesiologists, medical officers, nurses and auxiliary nurse-midwives). The number of human resources included part-time and fulltime staff regardless of the number of years they had been working at the facility. Obstetricians and anesthesiologists were defined as those doctors who had a postgraduate training in these specific fields. The category medical officers included non-specialist doctors trained in western or Indian systems of medicine. Nurses were defined as those individuals who had completed a 3–4 year program in nursing education. On the other hand, auxiliary nurses were those who had a one-year basic nursing training.

#### Analysis

Data were analyzed with Stata v12 (StataCorp, College Station, Texas). Categorical variables were reported as frequencies and percentages. Graphs were used to display the data when appropriate. Comparisons were made between public and private facilities using Chi^2^ test. Medians and interquartile rage (IQR) were used to describe the number of signal functions between “Less-than-BEmOC + CS’ and “Less-than-BEmOC” facilities (significant differences were assessed using Mann–Whitney *u* test). Differences between these groups were considered significant if *p* < 0.05.

The number of human resources (midwifes and doctors), the number CEmOC and BEmOC facilities in each district were compared against the 2005 World Health Report (WHR) benchmarks as described by Gabrysch et al [13]. The WHR 2005 report suggest at least 20 midwifes and three part-time doctors per 3600 births [13]. In addition, they state that at least one CEmOC facility and one to two BEmOC facilities per 3600 births are needed for optimal EmOC coverage [13].

We have chosen to use the WHR 2005 benchmarks as these take into consideration the number of births to indicate the number of facilities/ staff required in a particular area. This, it has been argued is a better predictor of maternal mortality than United Nations’ benchmarks, which suggest indicators of facility numbers based only on total population, but not birth rates [13]. In order to estimate the number of births in each district per year we multiplied the district population by its crude birth rate (CBR).

## Results

### Overall EmOC availability

EmOC provision in the three districts under study was dominated by private facilities (70.4 %, 112/159) (Table 2). 22 % (35/159) of all facilities providing childbirth care were located in rural areas while the rest were located in district headquarters or small urban towns (taluks). Forty four % (21/47) of all public facilities were located in rural settings whereas only 12.5 % (14/112) of private facilities were (Table 2). Of the 47 public facilities included in this study three were district hospitals, 32 were sub-district hospitals or community health centers and 12 were primary health centers.

**Table 2:** Geographical location, emergency obstetric care classification, and signal functions by facility ownership, *n* = 159

Characteristics	All		Public		Private	
	*n* = 159		*n* = 47		*n* = 112	
	n	%	n	%	n	%
District^a^						
Sabarkantha	76	47.8	14	29.8	62	55.4^b^
Dahod	42	26.4	21	44.7	21	18.7
Surendranaga	41	25.7	12	25.5	29	25.9
Geographical location^a^						
District headquarters	33	20.7	3	6.4	31	27.7^b^
Small town#	91	57.2	23	48.9	67	59.8
Rural	35	22.1	21	44.7	14	12.5
EmOC classification^a,c^						
CEmOC	23	14.4	3	6.4	20	17.9^b^
BEmOC	3	1.8	1	2.1	2	1.8
Less-than-BEmOC	61	38.4	40	85.1	21	18.7
Less-than-BEmOC+ C-section	72	45.3	3	6.4	69	61.6

^a^Column percentages. ^b^Comparison between public and private facilities, *p* < 0.0001. ^c^Each district is divided on average into 8–10 blocks. Small towns are sub-district towns (block or taluk towns) and are smaller than district headquarter cities. They are surrounded by approximately 100–150 villages in the block

The most common facility type was ‘Less-than-BemOC + CS’ facilities (45.3 %, 72/159). Almost all (95.8 %, 69/72) of the ‘Less-than-BemOC + CS’ were located in the private sector. “Less-than-BEmOC” were the second most common facility type, and most were located in the public sector (65 %, 40/61). CEmOC facilities were mostly private (20/23) (Table 2). There were only three BEmOC facilities in the districts, one of which was in the public sector. “Less-than-BEmOC facilities” were the predominant facilities in rural areas (30/35 - data not shown). The median number of signal functions was significantly higher in ‘Less-than-BemOC + CS’ facilities (7, IQR 6–8) than in ‘Less-than-BemOC’ facilities (3, IQR 2–4)(data not shown).

### Basic and comprehensive obstetric care signal functions by facility ownership

**Fig. 1:**
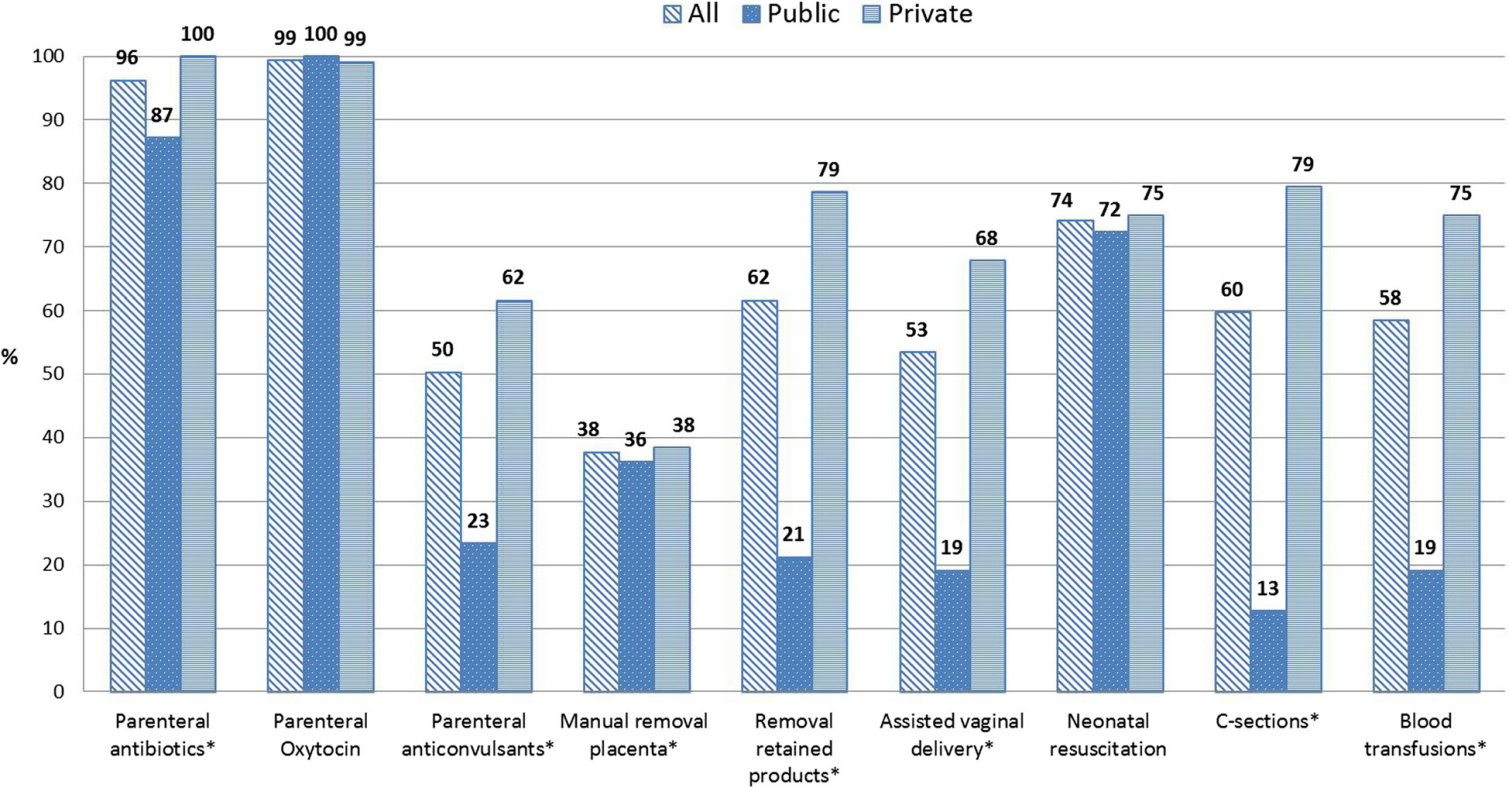
Percentage of emergency obstetric care signal function by facility ownership (* equals *p* < 0.0001)

Administration of parental antibiotics (96.2 %, 153/159), parenteral oxytocics (99.3 %, 158/159) and neonatal resuscitation (74.2 %, 118/159) were the three most often performed BEmOC functions. On the other hand, manual removal of placenta (37.7 %, 60/159), administration of parenteral anticonvulsants (50.3 %, 80/159) and assisted vaginal delivery (53.4 %, 85/159) were least performed (Fig. 1). Blood transfusions (93/159) and CS (95/159) were performed by six of every ten facilities (Fig. 1). Almost all functions (excluding neonatal resuscitation and administration of parenteral oxytocin) were significantly performed more often by private than public facilities (*p* < 0.001, Fig. 1).

### Reasons for non-performance of EmOC care signal functions

**Fig. 2:**
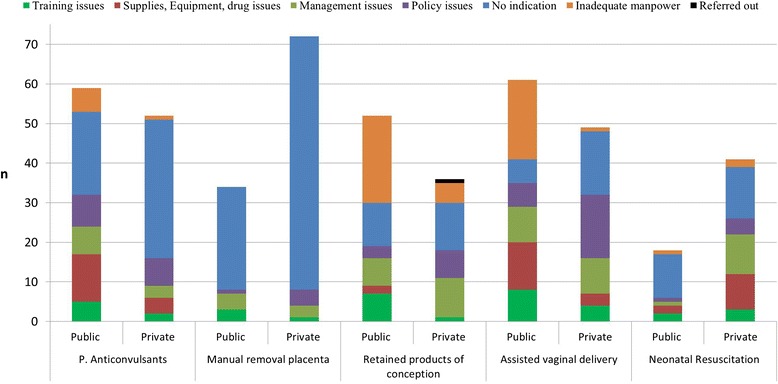
Number of reasons for not performing selected basic obstetric care signal functions by facility ownership

In both public and private facilities, “no indication” was the most common answer for not performing parenteral administration of anticonvulsants, manual removal of placenta or neonatal resuscitation (Fig. 2). In the public sector, “not having enough manpower” was the main reason for not performing removal of retained product of conception or assisted vaginal delivery. On the other hand, in private facilities “no indication” and “policy issues” were the most frequent reasons for no performing removal of retained products of conception and assisted vaginal delivery respectively (Fig. 2). In the public sector, “lack of supplies” was the most common reason for not having given blood transfusions, whereas “no indication” was the most frequent reason cited by private facilities (data not shown). For all facilities, “inadequate manpower” was the main reason for not performing CS (data not shown).

### Delivery loads and facility capacities

Delivery loads and human resources are described below. Seven of every ten mothers (20158/28303) delivered at private facilities. Most births in the private sector occurred at “less-than-BEmOC + CS facilities” (62 %, 12581/20158). Of public facility births, two thirds (5169/8145) occurred at “less-than-BEmOC facilities” (Table 3). Overall, 71 % (20077 /28303) of all institutional births occurred in facilities that had access to CS. 86 % (17275/20077) of all birth in facilities with access to cesarean sections occurred in private facilities. However, a quarter of all births (7722/28303) occurred in facilities with less than BEmOC capacity (and no CS). The number of beds available followed a similar distribution to the births described above (Table 3).

**Table 3:** Number of births in the last three months and beds stratified by EmOC facility classification and ownership

Facility type	Births						Beds					
	All		Public		Private^a^		All		Public		Private^a^	
	n	%	n	%	n	%	n	%	n	%	n	%
CEmOC	6821	24.1	2127	26.1	4694	23.3	924	22.2	246	15.2	678	26.7
Less-than-BEmOC+ C-section	13256	46.8	675	8.3	12581	62.4	1975	47.6	336	20.8	1639	64.5
BEmOC	504	1.8	174	2.1	330	1.6	72	1.7	56	3.5	16	0.6
Less-than-BEmOC	7722	27.3	5169	63.5	2553	12.7	1187	28.5	978	60.5	209	8.2
Total	28303	100.0	8145	100.0	20158	100.0	4158	100.0	1616	100.0	2542	100.0

^a^Comparison between public and private facilities, *p* < 0.001

### Inequalities in human resources distribution

**Fig. 3:**
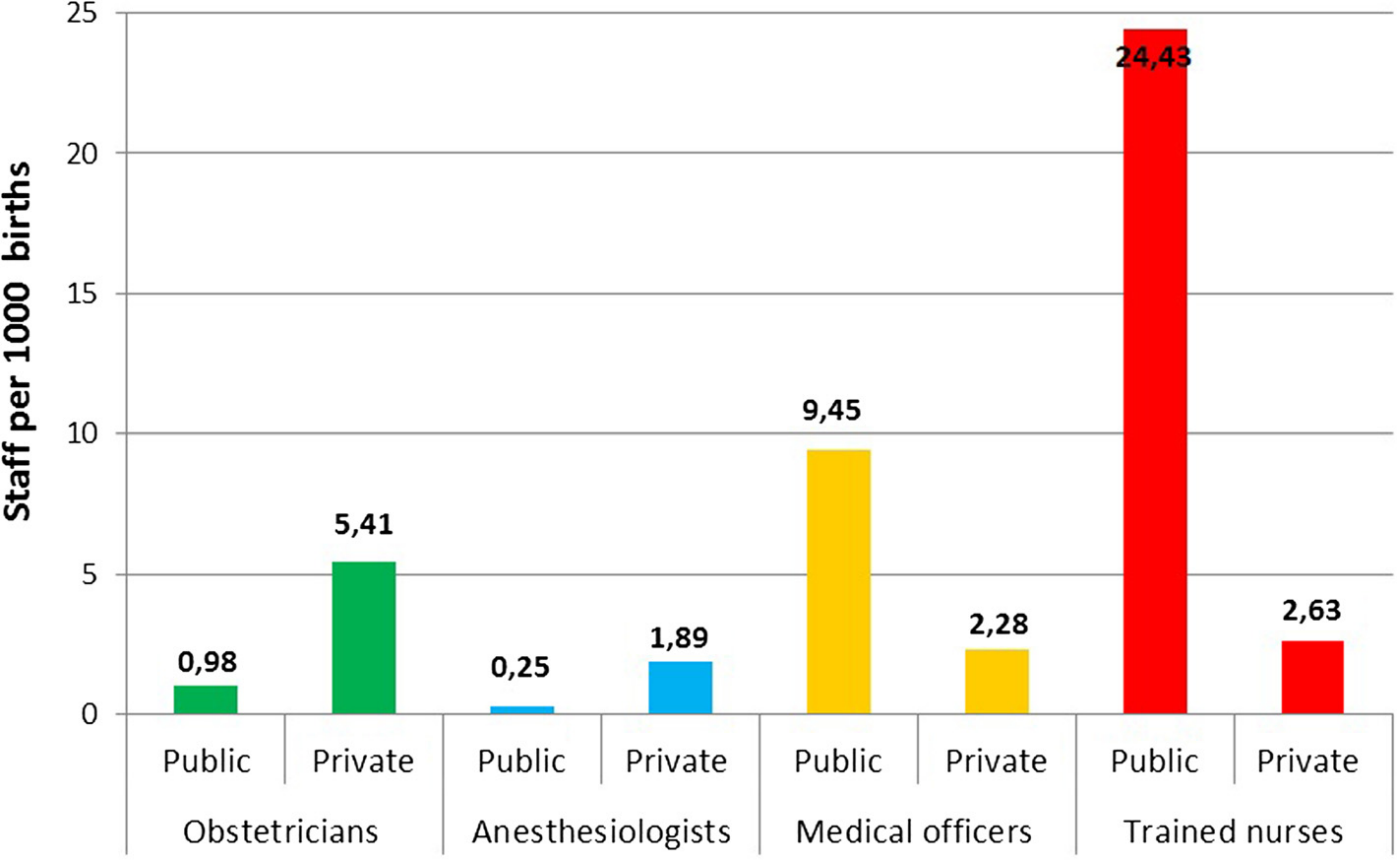
Specialists/physicians and nurses per 1000 births by facility ownership*

Inequalities in the availability of human resources are shown in Fig. 3. The rate of obstetricians and anesthesiologists per 1000 births working in the private sector was higher than those working in the public sector. On the other hand, the rate of medical officers (non-specialist) and trained nurses per 1000 births was higher in the public than in the private sector (Fig. 3).

### EmOC availability

EmOC availability in the study area by WHR 2005 benchmarks is described in Table 4. No district met the minimum required CEmOC facilities as set out by the WHR 2005 benchmarks. Dahod, the poorest district, had the largest deficiency. However, the number of facilities providing CSs but not all other eight obstetric care essential functions was high. The gap in the availability of BEmOC facilities was significant in all studied districts (Table 4). In all districts but one, the number of doctors exceeded the number required by the WHR 2005. However, wide gaps were found in the number of nurse-midwifes (Table 4).

**Table 4:** Staff and facilities per district compared against WHR 2005 benchmarks^a^

Indicator	Districts and number of births per year								
	Sabarkantha (births: 67,965)			Dahod (births: 64,222).			Surendranaga (births: 67,965).		
	Number of facilities or staff			Number of facilities or staff			Number of facilities or staff		
	Current	WHR 2005	Deficit	Current	WHR 2005	Deficit	Current	WHR 2005	Deficit
CEmOC	15	19	4	5	18	12	3	11	8
BEmOC	1	38	73	0	36	36	2	22	20
Less–than-BEmOC + C-sections	41	n.a	n.a	12	n.a	n.a	19	n.a	n.a
Midwives (nurses)^b^	103	378	275	98	357	259	51	224	173
Doctors (all)	126	57	0	55	54	1	59	34	0

^a^WHR 2005 benchmarks recommend the following: a.20 midwifes and three part-time doctors per 3600 births and b. one CEmOC facility and one to two BEmOC per 3600 births. ^b^Includes nurses and auxiliary nurses. Assuming all births delivered by this cadre

## Discussion

Our findings show that facilities and qualified obstetricians were largely in the private sector; which also accounted for three-quarters of all institutional births in our study. Facilities were clustered at two levels of function; (i) at CEmOC level and (ii) at less than BEmOC + CS level (71 % of all births occurred here). While the availability of CS was high, what was notable was the non-availability of BEmOC level facilities in either the private or public sectors.

### EmOC availability and distribution

The availability and distribution of EmOC facilities in our study was different compared to reports from sub-Saharan Africa [14, 15] and other parts of India [16], i e. the location of facilities with a qualified obstetrician and the potential to do a CS is not just restricted to large district headquarter cities, but also goes down to the level of smaller towns in community development blocks (small urban centers surrounded by approximately 100–200 villages). Also, the availability of qualified human resources (qualified obstetricians) is much higher than in sub-Saharan Africa; though in India the majority of these are in the private sector [11].

Although the wide availability of CS could be a strength, given that it is often lifesaving, it does raise questions of possible overuse of cesareans in this setting. Although more in-depth examination is necessary to look into reasons for CS particularly in the private sector, aggregate figures suggest this is unlikely as sub-national community based surveys [17] have shown that overall CS rates in Gujarat are low (6 %) despite the dominant private provision of obstetric care.

### Low performance of functions requiring manual skills in the public and private sectors

BEmOC functions requiring advanced manual skills (manual removal of placenta and assisted vaginal delivery) were among the least performed functions; this is in line with reports from South India [16] and Africa [15]. Several reasons might explain these findings. As reported in our results, there may not be enough cases seen in each facility to keep these skills alive. Staff may also lack training to perform these procedures [18] or may replace them with procedures they are more comfortable with performing (i.e. CS). For example, the inverse relation between CS and instrumental childbirth rates has been reported elsewhere [19]. Nevertheless, the attrition of skills from non-performance of procedures is of concern. Further studies are needed to identify the reasons behind the poor performance of these skills in this setting.

### The use of WHR 2005 benchmarks to assess the adequacy of EmOC

Different benchmarks have described and used to evaluate the sufficiency of the supply side of childbirth care for women [13]. Among those are the UN 2009 [1] and the WHR 2005 [8] benchmarks. Most studies looking at EmOC availability have used the UN 2009 benchmarks to assess the adequacy of facilities and staff in a given setting [20–22]. However, it has been argued that the WHR 2005 benchmarks are more useful than the UN 2009 benchmarks for planning and providing health services. The WHR 2005 benchmarks correlate better with maternal mortality and provide more consistent estimates across different levels of crude birth rates [13].

In our study, we have used the WHR 2005 benchmarks and we found that the numbers of BEmOC and CEmOC facilities were below these benchmarks [8], with the largest gap in BEmOC facilities. Our use of the WHR 2005 benchmarks make our findings difficult to compare with studies using the UN 2009 benchmarks. Nevertheless, the insufficient number of BEmOC facilities found in these studies and in ours is a consistent finding across many settings regardless of the benchmarking standard used [20, 21].

The inadequate number of BEmOC found in this setting is an issue of concern since it has negative consequences for mothers and health systems. Facilities that are unable to provide key obstetric services are more likely to be bypassed resulting in wasteful expenditure for the health system, delays in EmOC access, and overcrowding of higher level facilities [23]. Our study highlights that in this setting, EmOC care in the hinterland is provided mainly by less-than-BEmOC public facilities which restricts rural women’s access to adequate standards of obstetric care.

The insufficient number of CEmOC facilities in our study contrasts with studies conducted elsewhere reporting the opposite [14, 20, 22]. This might be explained by our use of the more strict WHR 2005 benchmarks [8] than UN 2009 benchmarks [1]. Thus, it is possible that studies reporting sufficient number of CEmOC facilities might have contrary findings if WHR 2005 standards were used [14].

### The role of Less-than BeMOC + CS facilities

Our study identified that in this setting the majority of births occurred at private facilities with the ability to do CS but unable to provide all other emergency obstetric care signal functions. Although these facilities did not perform all signal functions, they provided a median of seven comprehensive and key basic emergency obstetric care services (i.e., CS, blood transfusions, parenteral anticonvulsants, parenteral oxytocin, etc.) which cover the most frequently reported complications associated with maternal mortality in India [24]. Thus, these facilities can to some extent, compensate for the lack of fully functional BEmOC or CEmOC facilities in these districts.

### Human resources for EmOC

Our findings show that the rate of obstetricians and anesthesiologists per 100 births was significantly higher in the private than in the public sector. These numbers were lowest in the poorest (and more rural) district studied. This is cause for concern given that rural women in our study settings receive EmOC services mainly from the public sector. The shortage of specialized obstetric care in rural settings in India is commonplace [25] and highlights the deep inequalities in access to comprehensive care that rural women face.

All study districts met the WHR 2005 benchmarks [8] for doctors, but this was largely because of the presence of medical officers, a large majority of whom perform administrative tasks and are not necessarily practicing skilled birth attendants.

In our setting, nurses are often front line skilled birth attendants though their skills have been questioned [26]. It is also an issue of concern that their numbers fall below the WHR 2005 benchmarks, especially as they provide most intrapartum care in the public sector.

### Limitations

The generalizability of our findings to other parts the country should be done with caution given the heterogeneity that exists in other parts of Gujarat and the Indian Union. The level and availability of EmOC services even in our study districts varied widely. One important limitation of this and other studies evaluating the performance of signal functions at the facility level is that we cannot assert whether these services were provided to all women who needed them. Another possible limitation of our study is that we did not inquire into dual practice (public and private) by physicians and anesthesiologists. Nevertheless, given the large disparities in the distribution of these cadres between public and private facilities, it is unlikely that any overlap might significantly change the figures reported in our findings.

## Conclusions

We conclude that there is a strong provision of EmOC services in the private sector in the study districts of Gujarat but there is a need to focus on CEmOC provision in poorer districts. There is an under provision of functional BEmOC facilities, with some functions requiring manual skills being underperformed. This needs to be explored more in detail to ensure women get the appropriate services they need. Given the poor ability of the public sector to provide EmOC services, including the lack of appropriate human resources, the state needs to look into and strengthen innovative methods of covering this shortfall or developing partnerships with the private sector so that all women in the state receive the care they need, while strengthening the public sector.

## Abbreviations

BEmOC, basic emergency obstetric care; CEmOC, comprehensive emergency obstetric care; CS, cesarean-section; EmOC, emergency obstetric care; UN, United Nations; WHR, World Health Report
