# Erratum to: The dominance of the private sector in the provision of emergency obstetric care: studies from Gujarat, India

**DOI:** 10.1186/s12913-016-1559-3

**Published:** 2016-08-09

**Authors:** Mariano Salazar, Kranti Vora, Ayesha De Costa

**Affiliations:** 1 Department of Public Health Sciences, Karolinska Institutet, Tomtebodavägen 18a, Widerströmska Huset, 171 77 Stockholm, Sweden; 2 Department of Reproductive and Child Health, Indian Institute of Public Health, Gandhinagar, Ahmedabad, Gujarat India

## Erratum

Following the publication of this article [1] it was brought to our attention that two values in Table 4 were incorrect:

**Table 4:** Staff and facilities per district compared against WHR 2005 benchmarks^a^

Indicator	Districts and number of births per year								
	Sabarkantha (births: 67,965)			Dahod (births: 64,222)			Surendranaga (births: 67,965)		
	Number of facilities or staff			Number of facilities or staff			Number of facilities or staff		
	Current	WHR 2005	Deficit	Current	WHR 2005	Deficit	Current	WHR 2005	Deficit
CEmOC	15	19	4	5	18	13	3	11	8
BEmOC	1	38	37	0	36	36	2	22	20
Less–than-BEmOC + C-sections	41	n.a	n.a	12	n.a	n.a	19	n.a	n.a
Midwives (nurses)^b^	103	378	275	98	357	259	51	224	173
Doctors (all)	126	57	0	55	54	1	59	34	0

^a^WHR 2005 benchmarks recommend the following: a. 20 midwifes and three part-time doctors per 3600 births and b. one CEmOC facility and one to two BEmOC per 3600 births. ^b^Includes nurses and auxiliary nurses. Assuming all births delivered by this cadre

1. In the Sabarkantha district, under the Deficit column, number 73 should read 37.
2. In the Dahod district, under the Deficit column, number 12 should read 13.

## References

[1] Salazar M (2016). The dominance of the private sector in the provision of emergency obstetric care: studies from Gujarat, India. BMC Health Serv Res.

